# Skeletal muscle immobilisation-induced atrophy: mechanistic insights from human studies

**DOI:** 10.1042/CS20231198

**Published:** 2024-06-19

**Authors:** Colleen S. Deane, Matthew Piasecki, Philip J. Atherton

**Affiliations:** 1 Human Development and Health, Faculty of Medicine, University of Southampton, Southampton General Hospital, U.K.; 2 Centre of Metabolism, Ageing and Physiology (CoMAP), Medical Research Council/Versus Arthritis UK Centre of Excellence for Musculoskeletal Ageing Research (CMAR), National Institute of Health Research (NIHR) Biomedical Research Centre (BRC), University of Nottingham, U.K.

**Keywords:** atrophy, disuse, skeletal muscle

## Abstract

Periods of skeletal muscle disuse lead to rapid declines in muscle mass (atrophy), which is fundamentally underpinned by an imbalance between muscle protein synthesis (MPS) and muscle protein breakdown (MPB). The complex interplay of molecular mechanisms contributing to the altered regulation of muscle protein balance during disuse have been investigated but rarely synthesised in the context of humans. This narrative review discusses human models of muscle disuse and the ensuing inversely exponential rate of muscle atrophy. The molecular processes contributing to altered protein balance are explored, with a particular focus on growth and breakdown signalling pathways, mitochondrial adaptations and neuromuscular dysfunction. Finally, key research gaps within the disuse atrophy literature are highlighted providing future avenues to enhance our mechanistic understanding of human disuse atrophy.

## Introduction

Skeletal muscle atrophy, manifesting as a detectable loss of muscle mass, is the consequence of many communicable and non-communicable diseases such as ageing [1], diabetes [2], cancer [3,4] and chronic obstructive pulmonary disease [5,6]. In healthy (i.e., non-disease) states, the absence of muscle loading causes muscle atrophy, as is seen in the case of bed rest [7], unilateral lower limb immobilisation (ULLI) [8], unilateral lower limb suspension (ULLS) [9], reduced step count [10–12] and spaceflight [13]. This ‘non-disease’ associated muscle disuse atrophy is often referred to in the literature as ‘simple’ or ‘uncomplicated’ atrophy, whereby intrinsic processes, rather than systemic or external processes, result in the atrophy of the muscle(s) subjected to disuse [14,15]. Considering the structural (e.g. posture and locomotion [16]) and metabolic/endocrine (e.g. amino acid reservoir [17]) roles of skeletal muscles, the consequences of muscle atrophy are not trivial. As a result, muscle atrophy and the associated development of muscle weakness significantly increases the risk of frailty-related falls [18], morbidity [19] and mortality [20], while costing an estimated £2.5 bn annually in the UK alone [21]. Thus, muscle atrophy represents a significant and widespread socioeconomic burden.

Current countermeasures for muscle disuse atrophy include, but are not limited to, contractile activity, i.e., electrical stimulation [22,23], pre-habilitation [7], *in situ* resistance exercise [24] or nutrition, i.e., omega-3 supplementation [25,26], protein/amino acid supplementation [27,28] and structured feeding patterns [29]. Each, however, displays limited consistent efficacy in humans, while effective interventions remain elusive given our incomplete understanding of the underlying mechanisms of disuse atrophy [30]. In addition to non-pharmacological interventions, novel drug therapies targeting muscle atrophy (e.g. rapamycin and testosterone) are more recently being identified and explored [31]. As such, what is known about the metabolic and molecular changes that occur during disuse atrophy may help guide the design of effective human countermeasures. In lieu of this, the primary aim of this review is to summarise what is known regarding key mechanisms associated with immobilisation-induced atrophy. While previous reviews on disuse atrophy exist (e.g. [32–34]), this review focuses solely on the human literature of ‘simple/uncomplicated’ atrophy. Moreover, this review focuses on the mechanisms associated with disuse *atrophy*, not *dysfunction*, for which we direct the readers to the following resource [35].

## Human models of muscle disuse atrophy – bed rest/ULLI/S/step-reduction

**Figure 1:**
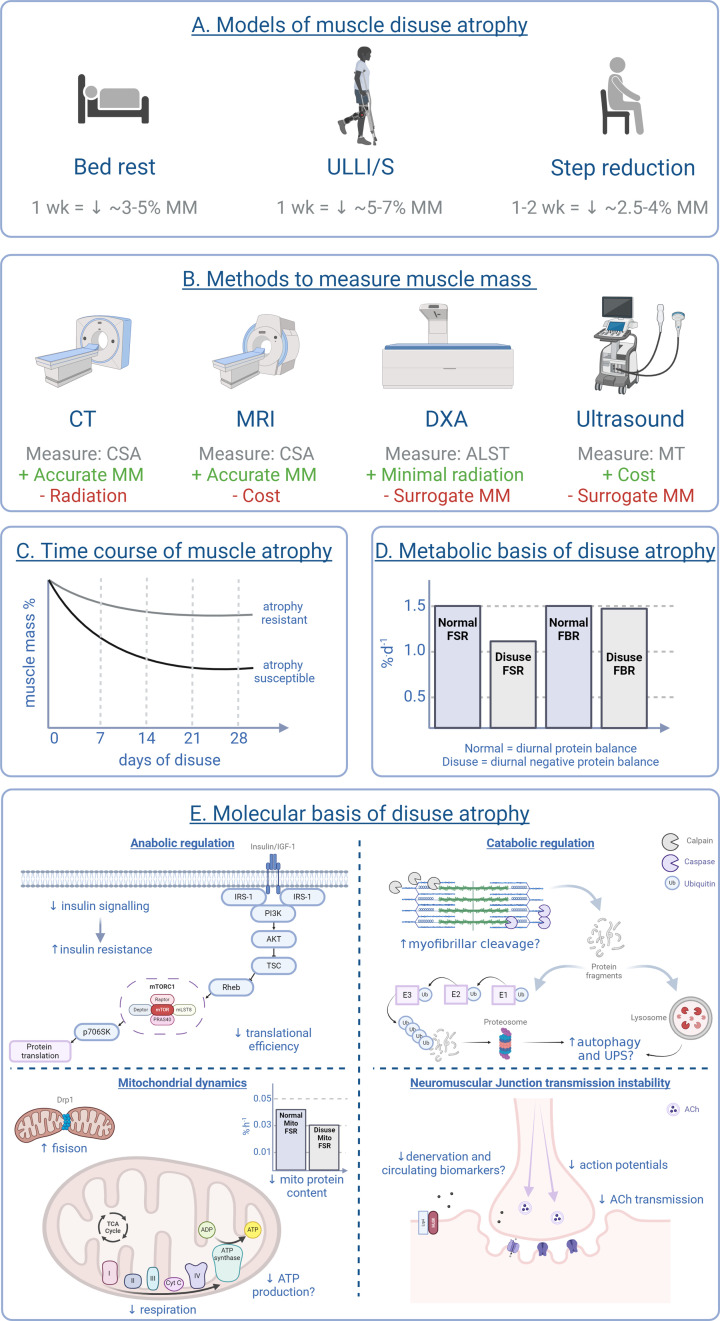
Summary of skeletal muscle disuse atrophy methods and mechanisms in humans (**A**) models of disuse muscle atrophy eliciting significant declines in leg muscle mass (**B**) methods to measure muscle mass often employed in human disuse studies, (**C**) time course of muscle atrophy, depicting the most rapid loss of muscle mass is within the first 14 days plateauing thereafter, (**D**) metabolic basis of disuse atrophy, depicting decreases in both fractional synthesis rate and fractional breakdown rate contribute to disuse atrophy, with the latter being of lesser magnitude, (**E**) molecular basis of disuse atrophy, demonstrating the contribution of anabolic signalling, catabolic signalling, mitochondria and neuromuscular junction dysfunction to disuse atrophy. Ach, acetylcholine; ALST, appendicular lean soft tissue; CSA, cross-sectional area; CT, computed tomography; Drp1, dynamin-related protein 1; DXA, dual-energy X-ray absorptiometry; E1, ubiquitin-activating enzyme; E2, ubiquitin-conjugating enzyme; E3, ubiquitin-ligase enzyme; FBR, fractional breakdown rate; FSR, fractional synthesis rate; mito, mitochondria; MM, muscle mass; MRI, magnetic resonance imaging; MT, muscle thickness; ub, ubiquitin; ULLI/S, unilateral lower limb immobilisation/suspension, UPS, ubiquitin proteosome system; wk; week; ‘+’ indicates a positive; ‘-’ indicates a negative. Created with Biorender.com.

Bed rest, ULLI (via casting) or ULLS (via bracing) and step count reduction are all key human experimental models of muscle disuse atrophy, which will be described and considered (Figure 1). Disease-associated models (e.g. denervation), radiation-models (e.g. spaceflight) and animal models (e.g. hind limb unloading) will not be considered here.

Experimental whole-body bed rest reflects periods of bed rest that occur alongside clinical scenario’s such as surgery, injury or illness. Experimental bed rest results in significant whole body lean mass declines of between 2.3 and 4.4% within a single week [36] due to the total amount of muscle tissue subjected to disuse and possibly due to whole body insulin resistance, systemic hormonal and/or inflammatory factors released [37]. In one specific bed rest study measuring the lower limbs, quadricep cross-sectional area declined by 3.2% in response to just 7 days bed rest, equating to 140 g of leg tissue lost, which was accompanied by an 8% decline in muscle strength [37]. Similar muscle mass and lean leg mass declines (∼3–5%) have been reported in a systematic review and meta-analysis of 25 bed rest studies [36].

Enforced ULLI/S is commonly a consequence of injury such as bone fractures or ligament tears. As an experimental model, ULLI/S isolates muscle disuse to a specific muscle group or single muscle while the contralateral leg serves as a control enabling a robust physiologic and molecular signal to be obtained within individuals [22,38]. As such, ULLI/S is considered an appropriate model to identify the mechanisms driving disuse-induced atrophy [37,38], which can induce muscle mass losses of up to 6.7% within 7 days of immobilisation [39]. In one specific study measuring the lower limbs, quadricep cross-sectional area declined by ∼5% in response to 7 days ULLI, equating to 220 g of leg tissue lost, which was accompanied by a 6% decline in muscle strength [37]. Similar muscle mass declines have been reported in a systematic review and meta-analysis of 86 single leg immobilisation studies [39].

Periods of reduced ambulatory activity (e.g. reduced step count) can occur in response to injury, illness and/or hospitalisation [40]. Reflecting this, step count reduction models typically reduce step count by ∼80% [11,41] or cap step count to 1,500 steps per day [10,12,40], which is recognised as a less severe model of muscle disuse compared with bed rest and ULLI/S. Despite the lower severity of the reduced ambulatory model, lean leg muscle mass significantly declines by ∼2.5-4% within 1–2 weeks [40,41], with declines of up to 9% seen in some individuals [40].

Considering that not all disuse protocols are equal regarding whole body muscle mass loss, systemic responses and organ responses, the model of choice must be considered carefully when designing and performing muscle disuse experiments and should reflect the key research question/s being asked and the real life application. In regard to ULLI/S, the amount of leg muscle tissue lost is similar when compared to bed rest (and in an internally controlled fashion) while also not evoking significant systemic and organ effects that are seen in bed rest (e.g. circulating factors and changes in cardiac output) [37]. Yet a common criticism of ULLI/S is that the weight bearing ‘control’ limb may support more of a person’s bodyweight during the disuse period incurring adaptation, therein not being a ‘true’ control. However, multiple independent studies have shown no change in muscle mass in the non-immobilised leg [35,38,42–44], indicative of no improved conditioning of the control limb. This is further supported by stable integrative MPS rates over a 7-day period in a non-immobilised limb, compared with declines in MPS rates in the immobilised limb [45]. It has also been argued that the weight bearing ‘control’ limb may bear less weight across the disuse period, a consequence of increased sedentarism due to casting or bracing. While muscle mass typically remains stable in the non-immobilised leg, there is some supporting evidence of deconditioning in the form of reduced vascular responses to sheer stress [46]. To overcome these potential cofounders, baseline muscle biopsies should be taken from both the non-immobilised and immobilised legs to allow temporal comparisons within and between limbs [38].

The bed rest model, which reflects periods of bed rest commonly experienced across a broad spectrum of clinical populations, is associated with a systemic inflammatory burden, cardiovascular deconditioning and bone loss. This may potentially affect the interpretation of results when trying to isolate the impact of immobilisation-induced atrophy. Therefore, the disuse model should be chosen based on the overarching research questions, the key outcomes sought and its intended application, while also considering the logistical and experimental challenges associated with each different disuse model.

## Methods to measure muscle mass

When comparing the quantity of muscle mass and rate of loss across different disuse scenarios, it is important to bear in mind that muscle mass can be measured in a variety of ways with differing levels of sensitivity. Computed Tomography (CT) is considered a gold-standard imaging techniques for quantitative analyses of skeletal muscle (cross-sectional area), also permitting the assessment of skeletal muscle ‘quality’, (i.e., fat infiltration into muscle) [47]. Although providing the most reliable measurements, CT comes at a great cost, requires expertise to conduct and subjects volunteers to ionizing radiation [47]. Magnetic Resonance Imaging (MRI) is a non-invasive imaging method used to quantify skeletal muscle mass (cross-sectional area) and ‘quality’, which is commonly adopted due to its excellent accuracy and reproducibility [48]. However, the associated costs and expertise remain key limitations associated with MRI, as does the need to exclude volunteers/patients based on certain implants [48]. Dual-energy x-ray absorptiometry (DXA) is a surrogate method to measure muscle mass, providing highly precise and accurate measures of lean soft tissue, with appendicular lean soft tissue being used for the estimation of muscle mass [49]. Despite DXA being a quick and relatively safe method using minimal radiation doses, it is costly, it requires expertise to conduct and the ability of DXA to reflect functional muscle mass is still questioned [49]. Unlike the other methods, ultrasound represents a cheap, user-friendly and relatively mobile method to robustly and reliably assess indices of muscle mass and architecture [50]. However, ultrasound is a surrogate measure using muscle thickness measurements for the estimation of muscle mass [51] and is subject to large experimenter variability [50]. Thus, while CT and MRI offer superior accuracy [52], DXA and ultrasound provide validated alternatives to estimate muscle mass (Figure 1). The varied use of these methods among the disuse literature makes direct comparisons of muscle atrophy challenging, particularly when trying to compare the rate of muscle atrophy across different disuse models.

## Time-course of muscle disuse atrophy

The rate of muscle disuse atrophy is inversely exponential, with the most rapid atrophy observed in the early disuse period, and gradually attenuating as the duration of disuse progresses (although inter-individual variability does exist [38,42]). Significant muscle atrophy has been detected as early as 2 days following disuse [42], with the greatest overall losses occurring within the first 14-day period, thereafter plateauing [53]. This biphasic response is true for all muscle groups regardless of the susceptibility or resistance to disuse atrophy (Figure 1). On this note, it is timely to raise the subject of individual muscle’s exhibiting distinct susceptibility to disuse atrophy [9]. For example, the triceps surae, an ‘atrophy susceptible’ muscle, displays ∼7% declines in muscle volume following 14 days of disuse, which increases (but does not double) to -11.2% by day 28, while the foot dorsiflexors, an ‘atrophy resistant’ muscle, displays a 1.8% decline in muscle volume following 14 days of disuse, which increases (but does not double) to -3.5% by day 28 [53]. Crucially thus, the biphasic temporality of muscle disuse atrophy implies different molecular mechanisms dominate at different periods of muscle disuse. Moreover, the mechanisms of muscle atrophy are likely to be different in magnitude in atrophy *susceptible* versus atrophy *resistant* muscles.

Readers are also directed to a recent systematic review and meta-analysis for greater insight into the rates and temporality of muscle disuse atrophy across muscle types [53]. While not the focus of this review, it’s worth noting that the rates of muscle atrophy can be 2.5 times greater in patients, i.e., in critically ill patients compared with healthy volunteers, due to the greater inflammatory burden and nutritional deficiencies [53].

## Metabolic muscle proteostasis and links to human disuse atrophy

In healthy, weightbearing adults, muscle mass is maintained via the balance between muscle protein synthesis (MPS) and muscle protein breakdown (MPB). These processes exist in a dynamic equilibrium driven by diurnal fasted-fed cycles, whereby dietary protein consumption replenishes amino acids liberated from muscle between feeding [54]. Disuse muscle atrophy is, therefore, the consequence of an imbalance between MPS and MPB, which is empirically attributed due to decreased MPS, increased MPB or a combination of both. That said: in humans the most likely scenario is decreases in *both* MPS and MPB, with the latter being of lesser magnitude [55].

Across disuse models, both postabsorptive (i.e. fasted) [54] and postprandial (i.e. fed) [54] myofibrillar MPS are reproducibly blunted, resulting in a 30–60% decline in overall MPS. The aforementioned blunted anabolic response to protein/amino acid feeding is known as ‘anabolic resistance’. MPB, on the other hand, either remains unchanged [54,56,57] or has been shown to decline [55,56], suggesting that bulk MPB does not appreciably contribute to disuse muscle atrophy in post-pubertal humans, which is also supported by mathematical estimates [58]. Deficits in fasted/fed MPS, as opposed to increases in MPB, should be considered the driving force of immobilisation-induced atrophy in humans in being sufficient in itself to explain muscle mass lost [57,59]. This may be caveated in that disease-associated muscle atrophy could be a consequence of decreased MPS and increased MPB due to prevailing systemic factors, e.g., circulating cytokines and cortisol, commonly associated with disease’s (e.g. [60,61]).

## Molecular proteostasis and links to human disuse atrophy

Despite the notion that decreases in MPS are the driving force in disuse atrophy, and that the underlying mechanisms remain incomplete, at the fore are deficiencies in translational capacity and/or efficiency [32,62]. During disuse, translational capacity (RNA:DNA ratio’s) seemingly remains unchanged [63], pointing toward altered translation efficiency as the dominant mechanism underpinning declines in MPS. The mechanistic target of rapamycin (mTOR) regulates translation initiation – the rate limiting step of protein synthesis – and is thus considered a key regulator of translational efficiency [32]. In response to disuse, mTOR and downstream signalling protein components (e.g. p70S6K) may exhibit reduced phosphorylation (e.g. [64]), although this is not a consistent finding, with others reporting no change in mTOR signalling despite reductions in MPS and muscle cross-sectional area [40,43,54]. While this dissociation between snapshot markers of anabolism and MPS may be explained by the chosen sampling timepoints missing the peak signalling changes, it also suggests that protein phosphorylation may not accurately proxy for MPS rates, as reported [15,65]. Considering mTOR hasn’t been consistently shown to exhibit reduced phosphorylation during disuse, this may indicate other signalling pathways as operative and potentially regulating MPS decline.

At the transcriptional level, a molecular gene network functionally related to ‘translational regulation’ has been shown to be up-regulated across disuse scenarios [62]. Although this may seem paradoxical given the protein level data, this increased expression is possibly a compensatory mechanism in response to disuse-induced declines in translational efficiency [62]. Highly connected ‘hub’ genes within this network purported to be crucial in driving this molecular response include *RLF Zinc finger* (*RLF*), which is necessary for embryonic muscle growth and is purported to increase methylation of factors associated with translational regulation [66], and *RING finger protein 13* (*RNF13*), a ubiquitin ligase implicated in muscle growth and involved in the post-translational modification of proteins [67]. These predicted genes represent candidate targets worthy of reverse translational investigation to determine their functional importance and therapeutic potential for regulating protein translation [62].

Transcriptional analysis has also identified the mTORC1/2 inhibitor, DEPTOR, as a highly connected hub gene of a disuse-activated molecular network, which positively correlates with declines in MPS (i.e. those with the greatest suppression in MPS display the greatest up-regulation of this network) [38]. While the exact mechanisms by which DEPTOR may inhibit MPS in human disuse scenarios is unknown, inhibition could theoretically occur at mTORC1, at the juncture of Akt and mTORC1 via PRAS40 or upstream of Akt via mTORC2 [38], supporting reduced phosphorylation levels of mTOR and downstream signalling protein components at the protein level (e.g. [64]). Nonetheless, this presents an interesting mechanistic avenue with potential therapeutic application worthy of investigation. Despite much of the muscle disuse atrophy literature to date focusing on elucidating the underlying regulatory anabolic mechanisms, research gaps remain (Table 1).

**Table 1:** Research gaps in understanding the mechanisms of muscle disuse atrophy in humans

Metabolic proteostasis	Molecular proteostasis	Mitochondrial dynamics	Insulin resistance	NMJ transmission instability
More direct MPB measurements	Detailed time course of activation/deactivation of mTOR signalling	Need temporal associative changes in bioenergetics and ETC complex content	Molecular drivers of insulin resistance	Require human data exploring full range of the motoneuron pool
Need for longer-term integrated rates of MPS	Identification of all upstream regulators of mTOR	Free-living mitochondrial protein turnover rates		Need integration of structural and functional impairment measurements
Use of dynamic proteomics to investigate single protein changes	Characterise the contribution of other anabolic signalling pathways to reduced MPS	Characterise mitochondrial ATP production rate measurements		Validation of denervation blood biomarkers
	Temporal calpain and caspase protein responses	Characterise mitochondrial cristae changes		
	Characterise autophagosome flux			
	Characterise the role of ubiquitin-activating (E1) and (E2) ubiquitin-conjugating enzymes			
	Contribution of UPS to bulk degradation versus modification of protein functions			
	Identification and characterisation of novel E1, E2 and E3 enzymes			
	Identify protein substrates of the UPS system			
	Characterise E3 turnover rates			

Despite the lack of bulk MPB (i.e. measured by stable isotope tracing and/or by inference from explanatory declines in MPS) being evident in humans, ‘targeted’ proteolysis regulating atrophy programming must remain a consideration since not all proteolytic activity may result in bulk MPB [32] and there is a known disassociation between MPB and snapshot molecular markers [65]. Within skeletal muscle, four main proteolytic systems have been identified as contributing to degrading the majority of cellular proteins and are, thus, implicated in disuse atrophy, which are calpains, caspases, lysosomes and proteosomes [68] (Figure 1).

Calpains are calcium-dependent cysteine proteases responsible for the cleavage of cytoskeletal/structural proteins (such as desmin, nebulin and titin), resulting in fragmented proteins that can be further degraded by other proteolytic systems [69]. Calpains are well recognised for their role in muscle remodelling, via the liberation of damaged myofibrillar proteins, paving the way for newer functional protein deposition. Despite the well-known role of calpains, their role and regulation in the context of disuse is vague. Calpains have been shown to increase [70], decrease [45,71,72] and remain unchanged [45,54,72,73] in response to periods of disuse, which is partly explained by which member of the calpain family has been measured. Early work showed gene expression of the ubiquitous calpains (i.e. calpain 1 and 2) and the calpain inhibitor, calpastatin, to remain unchanged following 2 weeks of immobilisation in healthy adults, suggesting ubiquitous calpains and calpastatin have limited, if any role, in muscle disuse atrophy [72], which has been supported by more recent work [54]. However, this isn’t a universal finding with similar studies reporting an increase in both calpain 1 and 2, advocating a role for ubiquitous calpains in the orchestration of disuse-induced myofibrillar dismantling [74]. While many facets of these studies were similar (e.g. age and sex), the muscle biopsies were taken from different muscles (*vastus lateralis* versus *gastrocnemius*), which may explain the divergent findings due to fibre type differences across the muscles [75]. The calpain family also contains the muscle specific calpain, calpain 3, which has been shown repeatedly to decrease in disuse scenarios [45,72,76]. The roles of calpain 3 are still being elucidated but one purported role is in regulating cell fate, whereby down-regulation of calpain 3 leads to the inactivation of NFκβ and increased cell apoptosis [72], which could contribute to atrophy progression. Despite the specificity of calpain 3 to muscle, beyond showing increases, there is a surprising lack of human data investigating its role in disuse atrophy. While no strong conclusions can be made regarding the role of calpains in human disuse atrophy without further temporal evidence, it is possible certain calpain family members contribute to ultrastructure remodelling via the cleavage of damaged/unwanted myofibrillar proteins.

Caspases are an additional family of proteases known for their role in apoptosis across multiple tissues [77]. Like calpains, caspases contribute to the initial liberation of myofibrillar proteins for degradation by other proteolytic systems. Despite the purported role of caspases, human evidence in disuse is sparse. Of the only known study, following 7 days of disuse caspase 3 mRNA was observed to increase, indicating the activity of this breakdown system. However, question marks remain over the temporal response of caspase 3, and the role of other caspases. In the absence of substantial evidence, the regulatory role of caspases (and calpains) in human disuse atrophy is unclear.

The autophagy-lysosome system is responsible for degrading and recycling bulk cytoplasm, long-lived proteins and organelles (e.g. ribosomes) [78,79]. The process of macroautophagy (referred to herein as autophagy) has several key steps, starting with initiation, which is the consequence of activated signalling pathways (i.e. mTOR and AMPK pathways). Following initiation, nucleation sees a small membrane structure begin to form in the cytoplasm, known as the isolation membrane or phagopore, which involves the unc-51-like autophagy activating kinase 1 complex (ULK1). This isolation membrane then elongates, engulfing cytoplasm in the process, to form a double-membraned autophagosome. This autophagosome fuses with a lysosome, which contains digestive enzymes, forming an autolysosome. Protein cargo tagged for degradation is then recognised and engulfed into the autophagosome, where it is degraded in the acidic lysosomal lumen by cathepsin proteases. Evidence suggesting autophagy significantly regulates disuse atrophy in humans is limited. Following 24 h of disuse, beclin-1, which is involved in autophagy nucleation, was shown to increase suggesting autophagosomes are being formed [80]. However, cathepsin L (a lysosomal protease) remained unchanged indicating no change in protease or degradation activity [80]. Considering the lack of evidence, no consensus exists on whether or not autophagy is activated and regulating human disuse atrophy due to the absence of multiple independent data sets. Future work should aim to quantify autophagosome flux alongside more comprehensive temporal protein content measures (Table 1).

The ubiquitin-proteosome system (UPS) is an ATP-dependent proteolytic system responsible for degrading fatefully tagged proteins [81]. The initial step in UPS-mediated degradation involves the binding of the small protein, ubiquitin, to the Ub-activating enzyme (E1), requiring ATP in the process [81]. Ubiquitin is then transferred from E1 to the Ub-conjugating enzyme (E2), and subsequently the Ub-ligase enzyme (E3) catalyses the conjugation of ubiquitin to the target protein [81]. This process iterates until at least four ubiquitin monomers are covalently linked to the target protein, which then forms the classical pattern recognised by the 26S proteasome as a signal to degrade the target protein [81]. In response to immobilisation-induced atrophy, proteolytic markers have most commonly been shown to increase [72–74,82–84], but have also been observed to decrease or remain unchanged [54] – possibly reflective of the duration of disuse (an argument we consider continues to cloud likely muti-phasic mechanisms). The most commonly investigated markers are the E3 ligases, MuRF1 and MAFbx/Atrogin-1, which were two of the first muscle-specific E3 ligases to be identified and the increased expression of these markers is traditionally thought to reflect bulk protein degradation [85], contributing to muscle atrophy. Considering that bulk MPB doesn’t increase in humans, as shown by tracer methods, it’s prudent to query the rise in proteolytic/UPS markers. One explanation is the known dissociation of metabolic snapshot/static markers with MPB (same being true for MPS) [65], suggesting that increases in MPB have been missed. However, recent integrated metabolic investigations showing stable MPB rates discredit this notion [54]. A more likely explanation of our contention is that rather than facilitating bulk protein breakdown, an early induction of UPS-related genes may impair protein synthetic capacity by, for example, ubiquitination of translation initiation-related proteins [32,54]. Indeed, this concept is in line with the rapid and significant decline in MPS seen in as little as 2 days of muscle disuse [45] and recent data quantitatively linking increased proteolytic-pathway gene expression to changes in MPS (i.e. greater expression in those with the greatest MPS depression) [38]. Ubiquitination is also not synonymous with degradation, resulting in many other functions such as protein trafficking via protein localisation and protein–protein interactions [85]. Based on this, we hypothesise that UPS induction in response to disuse may also indicate a manipulation in protein function, and not necessarily degradation and reduced protein abundance, hence no detectable decline in bulk MPB. It should be noted this does not dismiss a role for targeted proteolysis being an important mechanistic (but not explanatory) feature of human disuse atrophy. Nonetheless, while an increase in E3 markers may result in increased ubiquitination, the target protein substrates remain unknown [85], so it is unclear which proteins might be subjected to ubiquitination in response to disuse. This knowledge gap could soon be addressed since recent methodological strides have been made in identifying ubiquitinated proteins in response to exercise [86], which could be applied to disuse scenarios. It is also worthy to note that despite the existence of an estimated 600-700 E3 ligase genes [87], data is only available on a handful of these in human disuse scenarios, highlighting how little is understood about UPS-mediated degradation.

To summarise, our understanding of catabolic/proteolytic signalling in human disuse scenarios is scant relative to the number of studies in the area, with most investigating only a handful of markers using traditional wet lab techniques at limited time-points. It remains unclear whether monitoring changes in such catabolic markers represent appropriate surrogate measures of bulk protein degradation, but more than likely it does not. Many research gaps remain in this area, which we highlight in Table 1.

## Mitochondrial dynamics and links to human disuse atrophy

Mitochondria contribute to skeletal muscle homeostasis via multiple critical functions including ATP production, apoptosis, calcium handling, reactive oxygen species (ROS) regulation and antioxidant regulation. In healthy skeletal muscle, mitochondria account for ∼4–7% of cell volume and significantly correlate with maximal oxygen uptake [88]. As such, mitochondria intrinsically adapt to and reflect the metabolic demands of muscle tissue and are considered a key regulator of muscle disuse atrophy based upon pre-clinical evidence demonstrating the protective effect of mitochondria-related overexpression on disuse atrophy resistance [89]. Mitochondria serve as the site of ATP production via oxidative metabolism. Glucose is first oxidised into pyruvate during glycolysis, which is imported via the pyruvate transporter into the mitochondria where it is converted into acetyl coenzyme A (acetyl-CoA) [90]. Within the mitochondrial matrix, acetyl-CoA drives the citric acid cycle (TCA cycle, also known as the Krebs cycle), generating the electron carriers; nicotinamide adenine dinucleotide (NADH) and flavin adenine dinucleotide (FADH_2_), which donate electrons for mitochondrial oxidative phosphorylation [90]. The transfer of electrons across complexes I-IV of the electron transport chain (ETC) creates a proton gradient across the inner mitochondrial membrane that ultimately drives ATP production via ATP synthase, requiring oxygen in the process [90]. Defects in oxidative metabolism can impair ATP production, which is vital for protein synthesis and muscle function, and has thus been implicated in muscle disuse atrophy. In response to disuse, maximal and submaximal mitochondrial respiration is reduced independent of age [26,91–93], with the onset seen as early as 3 days following disuse, persisting at 14 days [26]. These reductions in respiration could theoretically reflect reduced ATP production, resulting in less ATP availability for MPS (Figure 1). This is supported by data displaying rapid declines in MPS rates in response to disuse (as previously discussed), although from the currently available literature blunted MPS and the ensuing atrophy may precede declines in mitochondrial respiration. In order to conclusively determine whether disuse-induced declines in mitochondrial respiration drive the declines in MPS and muscle atrophy, studies with high temporal resolution measuring these mechanisms, alongside direct ATP production rate measurements are needed [94].

While the mechanisms underlying reduced mitochondrial respiration may be multifactorial, independent studies have shown perturbed mitochondrial function-related transcriptional signatures [62,91,95] and reduced mitochondrial protein content [96]. Specifically, studies have repeatedly demonstrated reductions in citrate synthase (a marker of mitochondrial content) and ETC complex I-V protein content [93], indicating mitochondrial respiratory declines are possibly driven by reductions in mitochondrial content [91]. That said, respiratory decline has also been shown to precede declines in ETC proteins [26], possibly implicating rapid post-translational modifications of the ETC complexes as the regulatory mechanisms behind rapid declines in respiration [93]. Inconsistent findings in regard to the content of mitochondrial proteins may be explained by the estimated half-life of mitochondrial proteins of ∼14 days (albeit derived from studies in animals) [97], meaning that small changes in mitochondrial content may be hard to detect or easy to miss [93]. The inverse has also been found where disuse led to mitochondrial content declines but in the absence of respiratory decline [98]. In this case, it is plausible that post-translational moderations of ETC complex proteins transiently increase respiration [98], prior to respiratory decline [93]. Although in the absence of mitochondrial respiratory measurement, recent work in older adults demonstrated no changes in citrate synthase protein content or ETC complex I-V protein content [99] despite declines in muscle cross-sectional area and myofibrillar MPS rates [24]. This adds to the thesis that muscle atrophy precedes declines in mitochondrial content, at least in healthy older adults.

At the transcriptional level, molecular gene networks functionally related to mitochondrial function have been repeatedly shown to display reduced expression in response to disuse [62,100], indicative of functional mitochondrial changes. Recent transcriptional meta-analysis of multiple well-controlled disuse datasets identified highly connected hub genes predicted to be associated with the perturbed mitochondrial signature. Pertinent among the identified hub genes was *Cytochrome c oxidase subunit 4 isoform 1 (COX4I1*), which is an isoform of the electron transport chain complex IV, and a commonly employed marker of mitochondrial content and oxidative metabolism [101,102]. Considering that complex IV is the terminal electron acceptor contributing to the generation of the proton motive force that drives ATP production [103], it is possible that reduced *COX4I1* mechanistically contributes to the reduced mitochondrial respiratory capacity repeatedly observed following disuse. Other hub genes with purported mitochondrial-associated roles that were identified include *Glutamic-Oxaloacetic Transaminase 2 (GOT2*) and *Endonuclease G* (*ENDOG*). Thus, these transcriptional datasets offer candidate targets worthy of reverse translational investigation to determine their biological importance and therapeutic potential [62].

The now routine application of tracer measurements to determine mitochondrial protein synthesis rates, as previously done in the context of ageing [104], can also shed light on mitochondrial turnover in response to disuse. Indeed, following 14 days of disuse in older adults, mitochondrial protein synthesis rate was reduced [84], the likely consequence of a slower turnover of mitochondrial proteins. Functionally, this could result in mitochondrial dysfunction via the slower clearance of damaged mitochondrial proteins [84]. Similarly, following 7 days of disuse in young adults, postabsorptive and postprandial mitochondrial protein synthesis rates declined in the absence of mitochondrial respiratory decline [105]. This suggests that reduced mitochondrial protein synthesis precede and may contribute to reduced mitochondrial respiratory decline (Figure 1). However, respiratory decline has been observed as early as 3 days following disuse [26], but no corresponding mitochondrial protein turnover data are yet available at these early timepoints. Further work is needed to disentangle the cause or consequence relationship between mitochondrial respiratory and turnover changes.

As dynamic organelles, mitochondria can undergo remodelling processes to meet the metabolic demands of the tissue through fusion and fission. Under normal conditions, fusion and fission serve to dilute dysfunctional mitochondria, therein maintaining a healthy mitochondrial pool [90]. Fusion is the unification of two smaller mitochondria into a single larger mitochondrion, normalising mitochondrial membrane potential of damaged mitochondria by fusion with a healthy mitochondrion, therein also increasing TCA cycle activity and ATP production [90]. The process of fusion begins with the fusion of the outer mitochondrial membranes which is orchestrated by mitofusin-1 (Mfn-1) and mitofusin-2 (Mfn-2), followed by fusion of the inner mitochondrial membranes, which is orchestrated by optic atrophy-1 (Opa-1) [90]. Fission sees the fragmentation of mitochondria, whereby fission protein 1 (Fis1) orchestrates outer mitochondrial membrane fission [106] and dynamin-related protein 1 (Drp1) is recruited to the outer mitochondrial membrane where it constricts around the mitochondria to promote the division of the organelle [106]. Given that mitochondrial dynamics are implicated in regulating mitochondrial content which consistently declines in response to disuse [84,105], it is logical to hypothesise disuse-associated changes in fusion and fission processes. Indeed, following 3 and 14 days of disuse in younger adults the Mfn-2/Drp1 protein ratio was lowered, suggesting greater fission activation [26]. Considering fission is the removal of damaged mitochondria which is most likely destined for breakdown, this supports data showing lowered mitochondrial content in response to disuse (Figure 1). On the contrary, multiple studies have reported no change in protein markers of fusion and fission in response to disuse in older adults [26,99]. These discrepancies could be due to differing disuse time periods, sampling timepoints and age of volunteers. Moreover, as already highlighted, the turnover of mitochondrial proteins is estimated as ∼14 days, so it is plausible that changes in the proteins regulating these processes could have been missed. Nonetheless, temporal studies reporting fission and fusion are required to delineate dynamics during disuse.

Mitophagy is a mitochondrial quality control mechanism that functions to maintain mitochondrial homeostasis via the selective degradation of dysfunctional/depolarised mitochondria via the autophagy-lysosome system (which functions as previously described) [90]. This selective breakdown process is proposed to minimise excessive release of mitochondrial damage-associated molecular patterns and reactive oxygen species from damaged mitochondria [90]. Mitophagy is thought to regulate, at least partly, the disuse-induced declines in mitochondrial and ETC complex protein content [26]. Despite the assumed role, studies have reported no changes in ULK1 mRNA with 7 days of disuse in younger adults [107] or in ULK1 protein following 5 days of disuse in older adults [99]. Similarly, others have shown no protein content change in BCL2 interacting protein 3 (BNIP3), which regulates mitophagy [108], in older adults following 10 days of disuse [109]. The disassociation between declines in mitochondrial protein content with no apparent change in mitophagy markers suggests that decreased mitochondrial-related gene transcription [62] as opposed to mitophagy-related clearance is responsible for mitochondrial decline. It is also possible that, due to the sparse available evidence, mitophagy related dynamics have not yet been captured. In summary, mitochondria rapidly adapt to disuse evident by consistently observed reductions in mitochondrial oxidative capacity (respiration and content), which could be a cause or consequence of the atrophy phenotype. Whether declines in mitochondrial content precede functional decline, or vice versa, remains debatable. Despite the described mitochondrial changes as a cause/consequence of disuse atrophy, many aspects of mitochondrial biology remain vastly under researched (Table 1), such as mitochondrial calcium regulation, mitochondrial signalling and mitochondrial motility, which were not discussed herein due to the absence of data.

## Insulin resistance and links to human disuse atrophy

Skeletal muscle accounts for ∼80% of glucose uptake following feeding [14], thus contributing to metabolic health. However, in response to disuse, there is a rapid blunting in insulin-mediated glucose uptake [55,110,111] and the ensuing development of insulin resistance [93], at both the whole body and leg level [111]. This was convincingly demonstrated in healthy volunteers, where 7 days of bed rest led to reduced glucose infusion rate, reduced leg glucose extraction rate and lower content/activity of key proteins in glucose transport, phosphorylation, and storage [110]. Despite the undeniable onset of insulin resistance in response to disuse, these observations do not differentiate between being causative, or a consequence of disuse-induced atrophy.

The temporality of these metabolic responses provides insight into the regulatory mechanisms of disuse-induced insulin resistance. Within as little as 24 h of disuse, glucose sensitivity and tolerance declines [107], which develops to full-blown insulin resistance by 3 days [112], without accompanying lipid accumulation (or ceramide or phospholipid species content) [96,113] or increased mitochondrial ROS emissions in the presence of ADP [93]. Although longer periods of disuse (>4 weeks) are associated with significant intramyocellular lipid accumulation, no further declines in insulin sensitivity are seen beyond those observed within the first week of disuse [114,115]. Similarly, although maximal ROS emissions (i.e. in the absence of ADP) increase in response to disuse, this is likely not reflective of the *in vivo* situation [93]. This discounts a mechanistic role for intramuscular reactive lipid concentrations and ROS and suggests lack of muscle contraction *per se* is the key physiological driver for disuse-induced insulin resistance. This is likely mediated by attenuations in intracellular signalling pathways of glucose transport, GLUT4, to the plasma membrane to facilitate glucose translocation into the muscle cell [14,116,117]. Indeed, insulin signalling (e.g. IRS1 and AS160) and GLUT4 protein has been shown on multiple occasions to decrease in response to disuse [107,118,119] (Figure 1 and Table 1).

## Neuromuscular junction transmission instability and links to human disuse atrophy

The mechanisms of atrophy described herein are contingent upon muscle fibre depolarisation and contraction, which in turn relies on successful communication between motoneuron and muscle at the neuromuscular junction (NMJ). The NMJ has a highly specific role in the mobilisation of synaptic vesicles and the transfer of acetylcholine (ACh) to post-synaptic muscle fibre ACh receptors, and its peripheral location within muscle permits the exploration of functional biomarkers using electrophysiological techniques [120]. Moreover, this also renders it the only synapse capable of *in vivo* investigation in humans.

Depending on the model of disuse employed, descending neural drive and ACh transmission at the NMJ is reduced (reduced step count) or almost entirely removed (immobilisation, bed rest) (Figure 1). Two recent studies independently used intramuscular electromyography (iEMG) in human *vastus lateralis* following 10 [121] and 15 days [35] of ULLI/S. Estimations of NMJ transmission instability [122] during sustained contractions of 10 and 25% of maximum were unaltered by disuse, strongly suggestive of minimal adaptation of the NMJ. Although adding to the negligible data available in humans, these data reveal little of NMJs activated during higher level contractions, which may feasibly respond differently to disuse [123].

Histological imaging of the human NMJ poses technical challenges owing to its relatively low abundance in a typical muscle biopsy sample, and reliable imaging often necessitates the use of cadaveric or post-amputee specimens [124]. However, more targeted biopsy approaches do increase yield beyond standard techniques [125]. Direct imaging of pre- and post-synaptic regions of the NMJ following disuse are not available in humans, but more indirect markers clearly implicate NMJ dysfunction and probable denervation. Neural cell adhesion molecule (NCAM) is upregulated in denervated muscle fibres and is readily detectable with immunohistochemistry [126]. NCAM positive fibres increased following 3 [127], 10 [128] and 14 days [129] of bed rest, indicating probable partial denervation.

The importance of the agrin-MuSK-Lrp4 pathway in regulating de/re-innervation at the NMJ [130,131] has attracted interest in circulating biomarkers indicative of NMJ dysfunction [132,133]. With cleavage of the neural form of agrin, C-terminal agrin (CAF) fragments are released into circulation and can be quantified [133]. Plasma CAF concentration increased ∼19% following 10 days of bed rest [128] and approximately 5% following 10 days of ULLS [121]. Although blood biomarkers of NMJ structure/function are appealing, their true causative relationship has not yet been determined.

The possibility of NMJ dysfunction occurring as a consequence of fibre atrophy, rather than a cause, cannot be overlooked. Particularly pertinent in this context are the inextricable links between mitochondrial dysfunction (outlined above) and NMJ degeneration [134]. Mitochondria are abundant in the energy intensive presynaptic region of the NMJ and their selective degeneration may stem from retrograde signalling from muscle to nerve [135]. Clearly, data bridging electrophysiological (function) and imaging (structure) of the human NMJ are lacking and will require a multi-methodological approach in any future studies (Table 1).

## Conclusion and future directions

In humans, acute periods of muscle disuse, such as bed rest, immobilisation and sedentarism, results in significant and rapid declines in skeletal muscle mass, resulting from a complex interplay of several metabolic and molecular mechanisms. In non-disease disuse atrophy, depressed post-absorptive and post-prandial MPS largely, if not fully, explain the disuse-induced muscle mass loss. This in turn, may be exacerbated by fibre denervation and/or NMJ dysfunction, but human data are inconclusive. Although bulk MPB doesn’t increase, increased proteolytic activity may impair MPS via the degradation of translational machinery. Mitochondrial dysfunction is a robust feature of disuse atrophy, likely contributing in some degree to disuse-induced insulin resistance and muscle atrophy. Despite vast interest in understanding the mechanisms regulating muscle disuse – evident by the growing number of human disuse studies in this area – granular mechanistic detail of key regulatory processes (e.g. proteolysis and mitochondrial function) is still outstanding. To close this gap, we highlight what we believe are the key research gaps that should to be addressed in order to better understand the mechanisms of immobilisation-induced atrophy in humans (Table 1).

## Data Availability

Data sharing is not applicable due to the nature of the manuscript being a narrative review.

## Open Access

Open access for this article was enabled by the participation of University of Nottingham in an all-inclusive *Read & Publish* agreement with Portland Press and the Biochemical Society under a transformative agreement with JISC.

## Abbreviations

Acetyl-CoA: acetyl coenzyme A
ACh: acetylcholine
ADP: adenosine diphosphate
AMPK: AMP-activated protein kinase
ATP: adenosine triphosphate
BNIP3: BCL2 interacting protein 3
CAF: C-terminal agrin
COX4I1: cytochrome c oxidase subunit 4 isoform 1
CT: computed tomography
Drp1: dynamin-related protein 1
DXA: dual-energy x-ray absorptiometry
ETC: electron transport chain
E1: ubiquitin-activating enzyme
E2: ubiquitin-conjugating enzyme
E3: ubiquitin-ligase enzyme
FADH_2_: flavin adenine dinucleotide
Fis1: fission protein 1
iEMG: intramuscular electromyography
Mfn-1: mitofusin-1
Mfn-2: mitofusin-2
MPB: muscle protein breakdown
MPS: muscle protein synthesis
MRI: magnetic resonance imaging
mTOR: mechanistic target of rapamycin
MuRF1: muscle RING-finger protein-1
NADH: nicotinamide adenine dinucleotide
NCAM: neural cell adhesion molecule
NMJ: neuromuscular junction
Opa-1: optic atrophy-1
ROS: reactive oxygen species
TCA: citric acid cycle
ULK1: unc-51-like kinase 1
ULLI: unilateral limb immobilisation
ULLS: unilateral limb suspension
UPS: ubiquitin proteosome system
